# Functional display of bioactive peptides on the vGFP scaffold

**DOI:** 10.1038/s41598-021-89421-y

**Published:** 2021-05-12

**Authors:** Sharon Min Qi Chee, Jantana Wongsantichon, Lau Sze Yi, Barindra Sana, Yuri Frosi, Robert C. Robinson, Farid J. Ghadessy

**Affiliations:** 1 p53 Laboratory, A*STAR, 8A Biomedical Grove, Singapore, 138648 Singapore; 2 Mahidol-Oxford Tropical Medicine Research Unit, Mahidol University, Bangkok, Thailand; 3 School of Biomolecular Science and Engineering (BSE), Vidyasirimedhi Institute of Science and Technology (VISTEC), Rayong, 21210 Thailand; 4 Research Institute for Interdisciplinary Science, Okayama University, Okayama, 700-8530 Japan

**Keywords:** X-ray crystallography, Protein design, Biochemistry, Biophysics, Cancer, Molecular biology, Structural biology, Biologics, Sensors and probes, Biological techniques, Protein design

## Abstract

Grafting bioactive peptides into recipient protein scaffolds can often increase their activities by conferring enhanced stability and cellular longevity. Here, we describe use of vGFP as a novel scaffold to display peptides. vGFP comprises GFP fused to a bound high affinity Enhancer nanobody that potentiates its fluorescence. We show that peptides inserted into the linker region between GFP and the Enhancer are correctly displayed for on-target interaction, both in vitro and in live cells by pull-down, measurement of target inhibition and imaging analyses. This is further confirmed by structural studies highlighting the optimal display of a vGFP-displayed peptide bound to Mdm2, the key negative regulator of p53 that is often overexpressed in cancer. We also demonstrate a potential biosensing application of the vGFP scaffold by showing target-dependent modulation of intrinsic fluorescence. vGFP is relatively thermostable, well-expressed and inherently fluorescent. These properties make it a useful scaffold to add to the existing tool box for displaying peptides that can disrupt clinically relevant protein–protein interactions.

## Introduction

Bioactive peptides can be inserted into host protein scaffolds to generate functional chimeras suitable for imaging, biosensing and therapeutic applications. An ideal scaffolding protein is typically small, monomeric, free of internal disulfide bonds and post-translational modifications, and thermostable. Some or all of these features prevail in described protein scaffolds including monobodies, knottins, affimers, DARPins, lipocalins, oxidases, adhirons and kunitz domains^1–9^. Many of these scaffolds can be used to create libraries displaying randomized peptides grafted into one or more permissive loop region(s). Subsequent library interrogation, often in the context of a directed evolution campaign, can lead to selection of high affinity binders to a target protein of interest. In this case, the selection process identifies optimally displayed peptide motifs that must both engage the target and be compatible with overall scaffold integrity. In comparison, grafting of known bioactive peptides into a scaffold protein does not necessarily generate efficient binders. Often, iterative modifications are required to address insert peptide length, site of peptide integration (where more than one site exists in the scaffold) and composition/length of flanking linker residues^10^. In some cases, use of a more compatible alternative scaffold that can mitigate the often destabilizing influence of peptide inserts must be explored^11–13^. Therefore, as any one scaffold does not necessarily fit all peptides efficiently, there is a continued requirement for novel alternatives to complement the existing tool box^13^.

Here, we describe use of the vGFP protein as a robust scaffold for displaying peptides. vGFP comprises super-folder GFP (sfGFP) fused C-terminally to an Enhancer nanobody. Binding of the nanobody to sfGFP subtly modulates its chromophore environment to yield a fusion protein displaying ~ 50% increased fluorescence over sfGFP alone^14,15^. Insertion of a p53-derived peptide into the linker region connecting the sfGFP and the Enhancer components facilitates binding of the modified vGFP to the key p53 negative regulator Mdm2 that is over-expressed in certain cancers^16,17^. We demonstrate use of the engineered vGFP (vGFP-M2) for in vitro biosensing and cellular Mdm2 inhibition and imaging. Crystallographic analysis of the vGFP-M2-Mdm2 complex provides further insight into target engagement by peptides grafted into this novel scaffold. We additionally use vGFP to present a peptide binding the translation initiation factor eIF4E, and demonstrate on-target cellular interaction.

## Results

### In vitro binding of engineered vGFP variants to Mdm2

**Figure 1:**
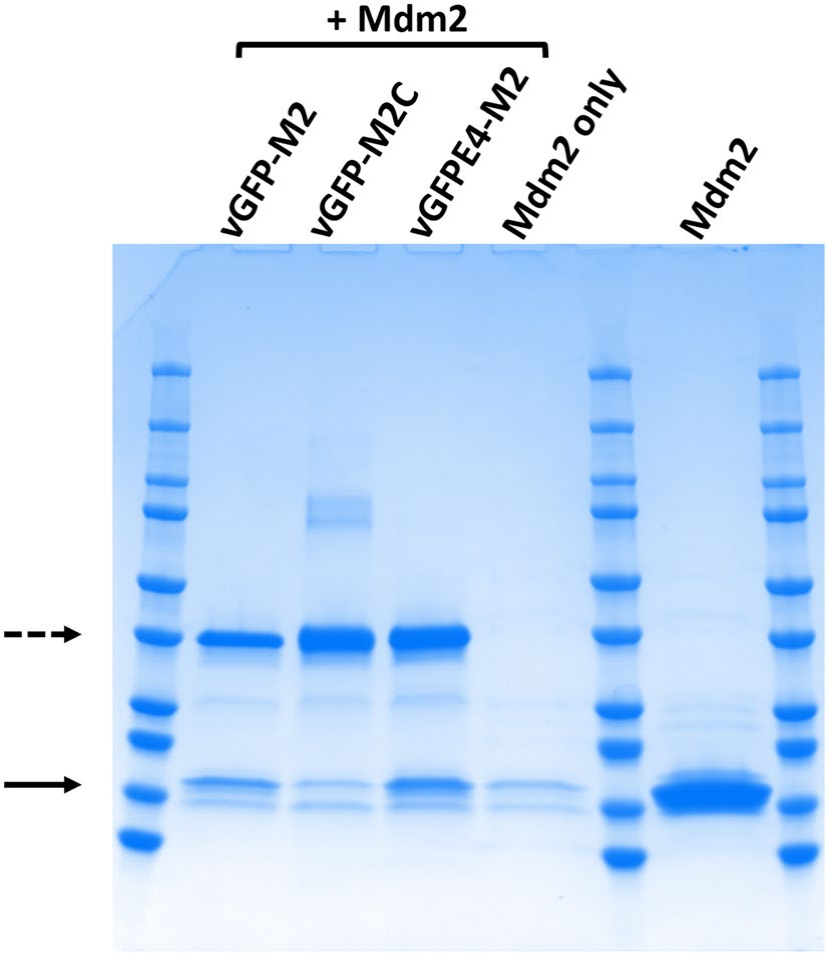
In vitro interaction of vGFP-scaffolded peptides with Mdm2 N-terminal domain (residues 6–125). Indicated purified proteins were (co)-incubated and the pulled down complexes analysed by SDS PAGE. Upper and lower arrows correspond to engineered vGFP proteins and interacting Mdm2, respectively. Experimental repeat is shown in Supplementary Fig. S1.

We first inserted a peptide sequence for a high affinity Mdm2 binder (Mtide-02, hereafter referred to as M2)^18^ in-between sfGFP and the Enhancer to generate vGFP-M2 (Table 1). In the corresponding negative control (vGFP-M2C) three of the M2 residues essential for high-affinity interaction with the N-terminal domain of Mdm2 were mutated to alanine^19^. Recombinant His-tagged vGFP-M2 and vGFP-M2C proteins were co-incubated with Mdm2 N-terminal domain protein (residues 6–125, hereafter termed Mdm2) and the complexes formed were pulled-down and analysed by SDS-PAGE. This assay showed clear interaction of vGFP-M2 with Mdm2, with background levels of binding seen for vGFP-M2C (Fig. 1).

**Table 1:** vGFP peptide-displaying constructs used in this study.

Construct	Sequence
vGFP-M2	sfGFP-VTAAGIT**TSFAEYWALLS**VQLVESG-Enhancer
vGFP-M2C	sfGFP-VTAAGIT**TSAAEYAALAS**VQLVESG-Enhancer
vGFP-M2.1	sfGFP-VTAAG**TSFAEYWALLS**VQLVESG-Enhancer
vGFP-M2.2	sfGFP-VTAA**TSFAEYWALLS**VQLVESG-Enhancer
vGFPE4-M2	sfGFP-VTAAGIT**TSFAEYWALLS**VQLVESG-Enhancer (S273A, R275A, S299A and F342A)
vGFP2-M2	sfGFP-VTAAGIT**TSFAEYWALLS(G**_**4**_**S)**_**2**_-VQLVESG-Enhancer
vGFP2-M2C	sfGFP-VTAAGIT**TSAAEYAALAS(G**_**4**_**S)**_**2**_-VQLVESG-Enhancer
vGFP3-M2	sfGFP-VTAAGIT**TSFAEYWALLSVQLVE(G**_**4**_**S)**_**2**_-VQLVESG-Enhancer
vGFP3-M2C	sfGFP-VTAAGIT**TSAAEYAALASVQLVE(G**_**4**_**S)**_**2**_-VQLVESG-Enhancer
vGFP-e4pep	sfGFP-VTAAGIT**RIIYDRKFMLECRN**VQLVESG-Enhancer
vGFP-e4pepC	sfGFP-VTAAGIT**RIIADRKFAAECRN**VQLVESG-Enhancer
vGFP-GSe4pep	sfGFP-VTAAGIT**GSRIIYDRKFMLECRN**VQLVESG-Enhancer
vGFP-GSe4pepC	sfGFP-VTAAGIT**GSRIIADRKFAAECRN**VQLVESG-Enhancer

Amino acids in bold depict peptide sequences grafted in linker region between sfGFP and the Enhancer components of vGFP.

Underlined residues in vGFP-M2, vGFP2/3-M2, vGFP-e4pep and vGFP-GSe4pep are mutated to alanine in respective non-binding controls (vGFP-M2C, vGFP2/3-M2C, vGFP-e4pepC and vGFP-GSe4pepC).

### In vitro biosensing of Mdm2 by vGFP variants

**Figure 2:**
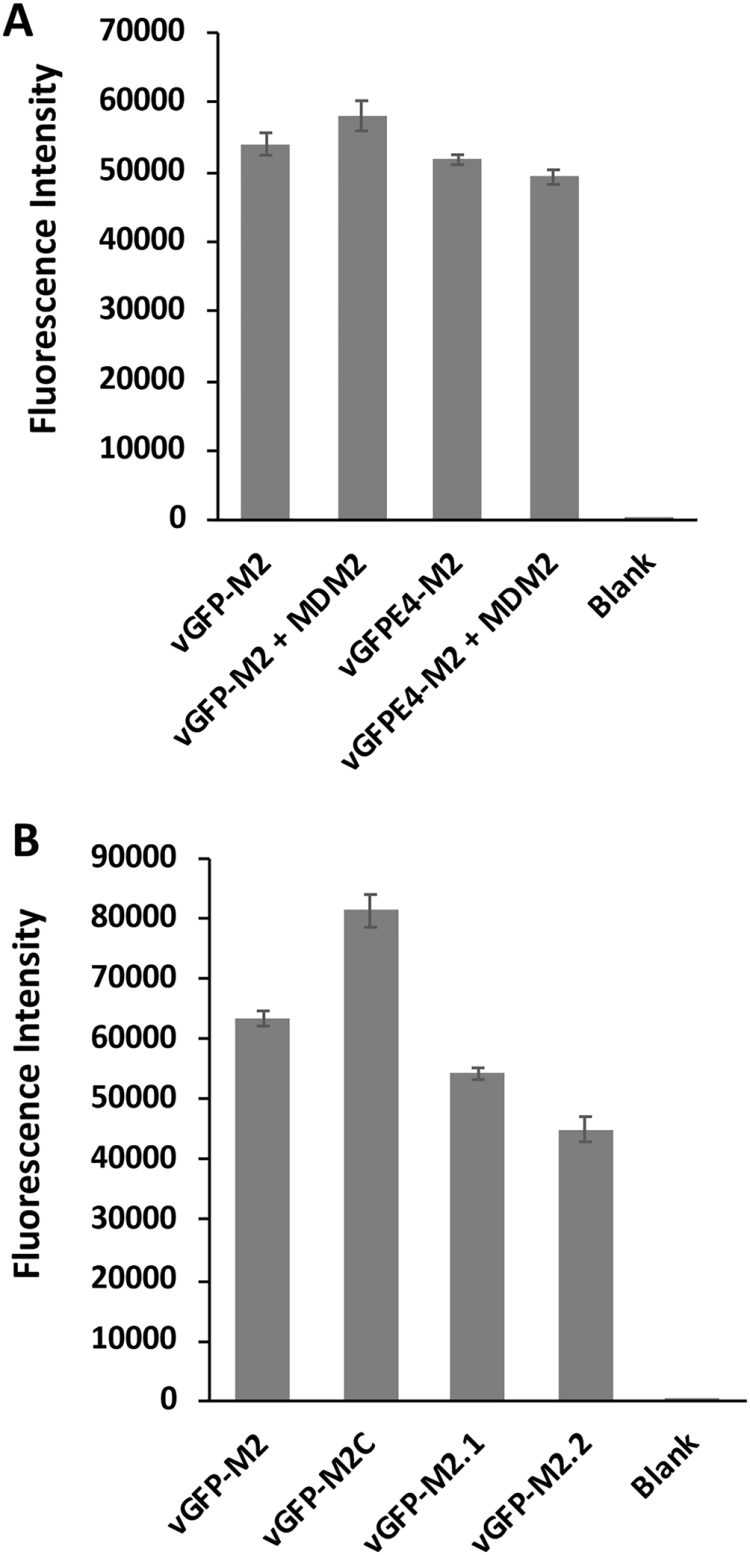
Fluorescence measurements of the engineered vGFP proteins. (**A**) The indicated vGFP proteins were incubated alone (0.23 µM) or in the presence of Mdm2 (6–125) (2.3 µM) and fluorescence measured. n = 2 ± SD. (**B**) Fluorescence measurement of the indicated vGFP proteins (0.25 µM). n = 2 ± SD.

p53 and Mdm2 have been used as a model interacting protein pair in the development of several biosensing technologies^20–25^. We have previously described a biosensor comprising β-lactamase coupled to an inhibitory protein (BLIP) in cis via a linker containing the M2 peptide sequence^26,27^. Upon binding to Mdm2, the normally disordered M2 peptide adopted an α-helical conformation, effectively shortening linker length and allosterically displacing BLIP and restoring β-lactamase activity. To test this allosteric derepression paradigm in vGFP-M2 we measured its fluorescence in the presence of Mdm2. No significant drop in fluorescence was seen (Fig. 2A), suggesting that any induced change in M2 conformation upon Mdm2 binding could not propagate sufficiently to displace the Enhancer from sfGFP. Crystallographic analysis of the complex confirmed this to be the case (see below). To improve the likelihood of displacing the Enhancer from sfGFP upon Mmd2 binding we shortened the linker region by removing either one or two residues from the disordered C-terminus of sfGFP (vGFP-M2.1 and vGFP-M2.2 respectively) (Table 1). However, in the absence of Mdm2 notable reduction in GFP fluorescence was observed when compared to vGFP-M2 (15% for vGFP-M2.1 and 29% for vGFP-M2.2) (Fig. 2B). This suggested that even minimal reduction in linker length significantly reduced the Enhancer-GFP interaction in cis, thereby limiting biosensing utility through measurement of fluorescence changes upon Mdm2 binding.

**Figure 3:**
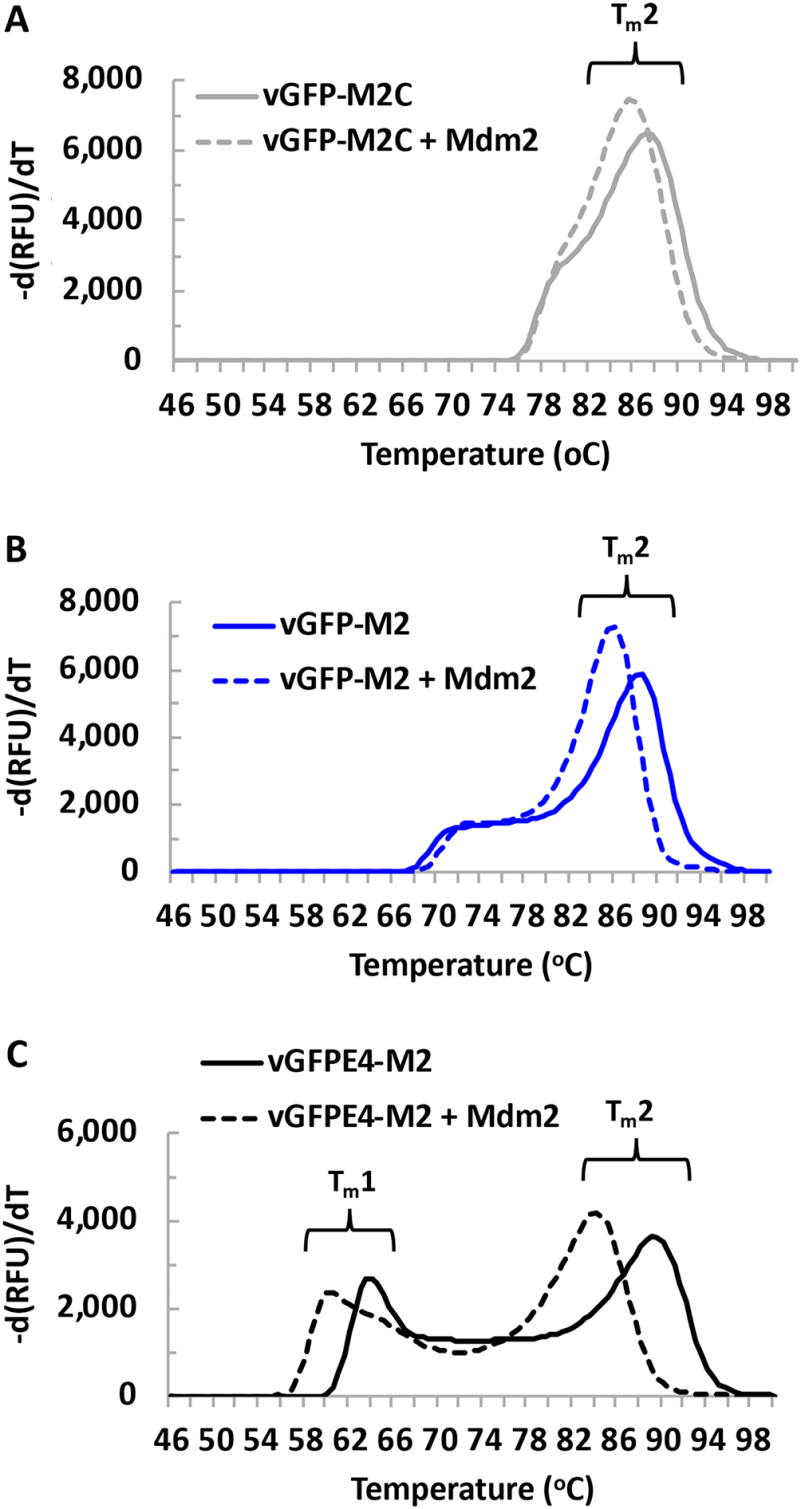
Thermal melt analysis of engineered vGFP proteins. The indicated vGFP variants were incubated alone (10 µM) or with Mdm2 (6–125) (100 µM) and fluorescence measured over a temperature range. Repeat experiments (n = 2) are shown in Supplementary Fig. S2.

**Figure 4:**
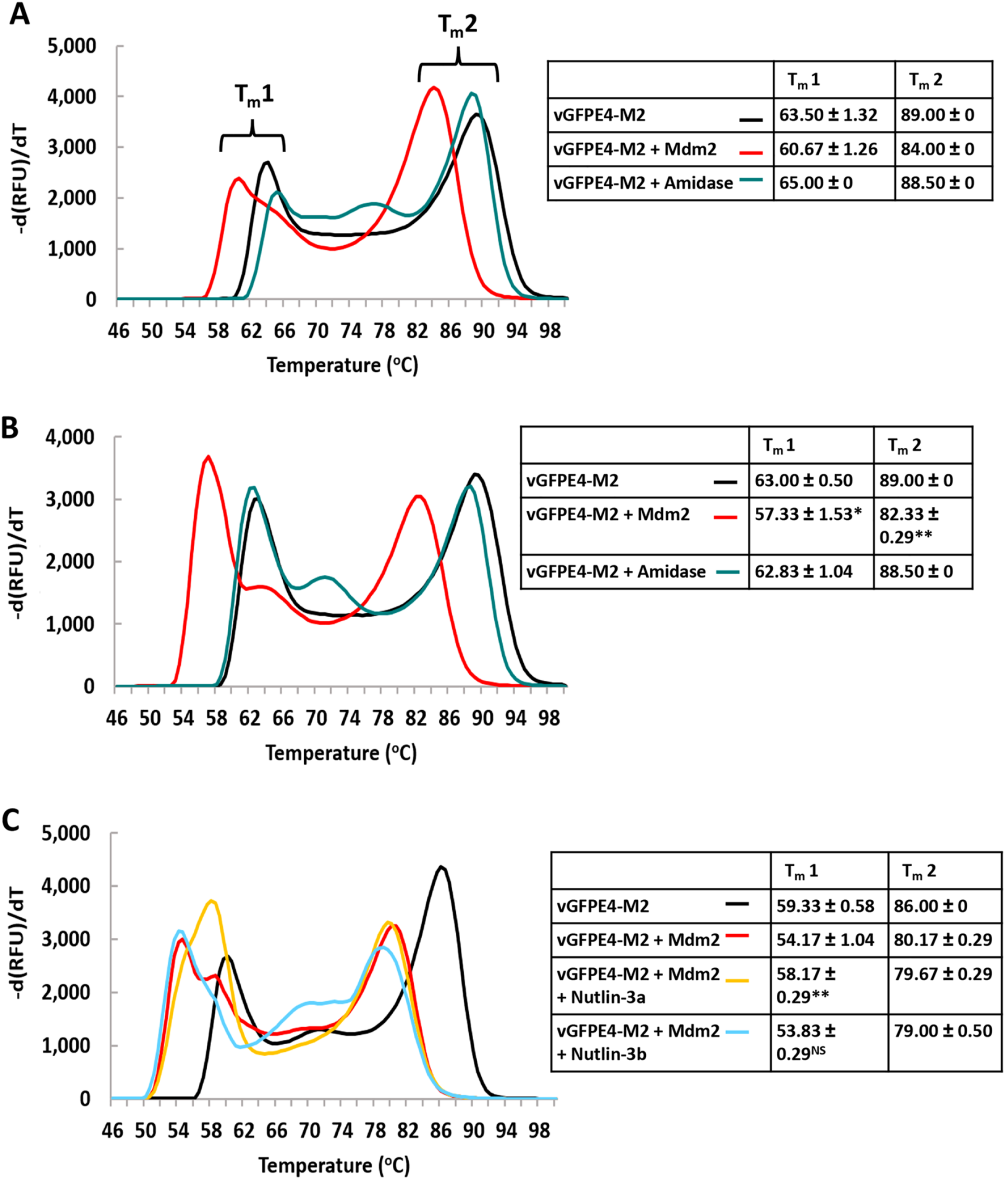
Specific detection of Mdm2 by vGFPE4-M2. (**A**) Melt curve analysis of vGFPE4-M2 alone (10 µM) or in the presence of specific (Mdm2) and non-specific (amidase) binders (100 µM). n = 3 ± SD. (**B**) Same as in **A** with addition of DMSO (10% v/v) in reaction mix. *p < 0.005 compared to T_m_1 of vGFPE4-M2 alone. **p < 0.0001 compared to T_m_2 of vGFPE4-M2 alone (Student’s t-test). (**C**) Melt curve analysis of vGFPE4-M2 (10 µM) in the presence of Mdm2 (100 µM) and specific (Nutlin 3a) and control (Nutlin 3b) small molecule competitors (1 mM). **p < 0.005 compared to T_m_1 of vGFPE4-M2 + Mdm2 and vGFPE4-M2 + Mdm2 + Nutlin 3b. ^NS^Not significant compared to T_m_1 of vGFPE4 + Mdm2 (Student’s t-test).

We therefore mutated 4 key residues in the Enhancer domain contributing towards its strong interaction with sfGFP to reduce its affinity^14^. This variant (vGFPE4-M2: S273A, R275A, S299A and F342A) maintained interaction with Mdm2 in a pull-down assay (Fig. 1). In the absence of Mdm2, vGFPE4-M2 fluorescence was the same as for vGFP-M2, indicating that the mutations did not abrogate fluorescence enhancement by the Enhancer. Incubation of vGFPE4-M2 with Mdm2 did not however result in any fluorescence change (Fig. 2A). We reasoned that differences in the intramolecular affinity of the Enhancer for sfGFP would become more evident at higher temperatures. Thermal melt curve analysis was therefore carried out on vGFP-M2, vGFP-M2C and vGFPE4-M2, using the intrinsic sfGFP fluorescence to report on protein stability and conformation. All three proteins showed a higher temperature melt peak (T_m_2, ~ 88 °C) corresponding to denaturation of the GFP chromophore (Fig. 3, Supplementary Fig. S2). Notably, a clearly distinct lower temperature melt peak (T_m_1, ~ 63 °C) was only observed for vGFPE4-M2. This is likely due to thermally induced intramolecular dissociation of the mutated Enhancer from GFP, resulting in decreased fluorescence. The corresponding event in the vGFP-M2/M2C proteins takes place at higher temperatures, represented by less distinct peaks at ~ 73 °C and ~ 78 °C respectively. Co-incubation with Mdm2 resulted in a clear downward shift of T_m_2 for vGFP-M2 and both T_m_1 and T_m_2 for vGFPE4-M2 (Fig. 3B,C, Supplementary Fig. S2). The melt profile of the non Mdm2-binding vGFP-M2C protein showed less difference in the presence of Mdm2 (Fig. 3A, Supplementary Fig. S2). Incubation of vGFPE4-M2 with a non-binding protein control (amidase) did not alter the melt profile, confirming an Mdm2-specific phenotype (Fig. 4A). The Mdm2-dependent shifts were further down-shifted in the presence of DMSO (10% v/v), an additive solvent that can modulate protein structure and interactions^28^ (Fig. 4B). Binding specificity was further validated using Nutlin 3a, a small molecule Mdm2 inhibitor that competes for binding to the same region of Mdm2 targeted by the scaffolded M2 peptide^29^. In the presence of Nutlin 3a the T_m_1 peak drifted towards that of vGFPE4-M2 alone. Intriguingly, the T_m_2 peak remained unchanged (Fig. 4C). The control Nutlin 3b enantiomer (with ~ 150-fold reduced binding affinity for Mdm2) did not have any notable effect on either melt peak. Nutlin 3a or 3b alone did not significantly affect the vGFPE4-M2 melt profile (Supplementary Fig. S3). Whilst these results indicate the potential for biosensing using vGFP, further engineering will be required to improve both sensitivity and robustness.

### Structure of vGFP-M2 bound to Mdm2

**Figure 5:**
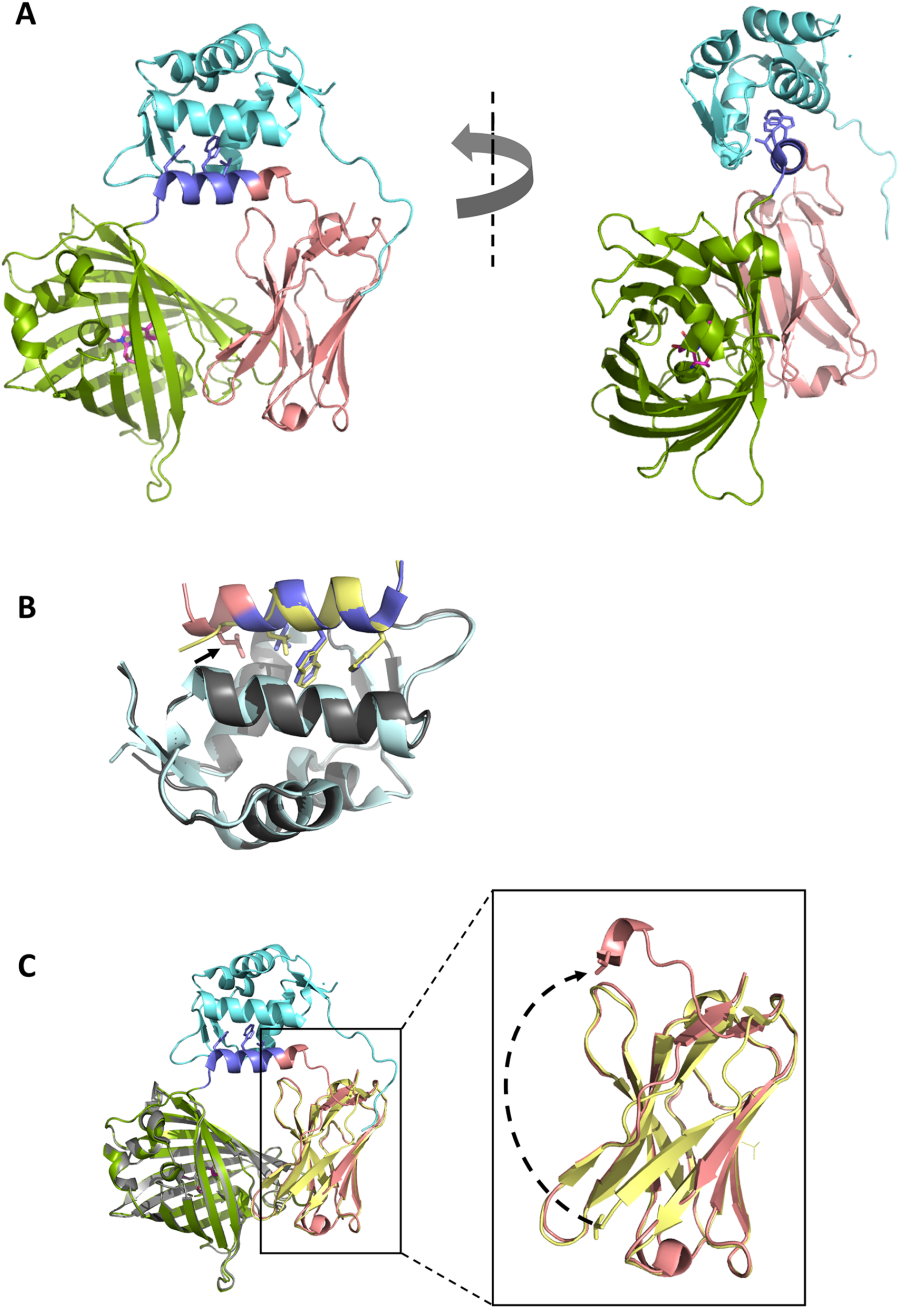
Crystal structure of vGFP-M2 bound to Mdm2 (6–125). (**A**) Structure of Mmd2 (6–125) bound to vGFP-M2. The sfGFP, Enhancer and M2 peptide components of vGFP-M2 are respectively coloured green, salmon and slate. Sidechains of Phe, Trp and Leu residues of M2 that project into Mdm2 (cyan) hydrophobic groove are depicted as sticks. (**B**) Structural overlay of vGFP-M2-Mdm2 structure (PDB ID: 5WTS) with structure of linear p53-binding peptide (yellow) bound to Mmd2 (grey) (PDB ID: 1YCR). Conserved Phe, Trp, and Leu side chains are depicted as sticks. Arrow highlights the Leu244 sidechain of the Enhancer (salmon) that also projects into Mdm2 hydrophobic groove. (**C**) Structural overlay of vGFP-M2-Mdm2 complex (same colouring as in (**A**)) and vGFP (grey) bound to the Enhancer (yellow) (PDB ID: 3K1K). Right panel highlights alternative conformations of the Enhancer N-terminal residues 1–10 in the two structures.

The biosensing data highlighted the need to further understand the linker conformation and interfacial Enhancer-sfGFP interactions in ligand-bound vGFP sensors. To this end, we next determined the crystal structure of vGFP-M2 bound to the Mdm2 N-terminal domain (residues 6–125). A single binary complex was present in the asymmetric unit, with the M2 peptide linker presented as an α-helix projecting three key residue sidechains (Phe, Trp and Leu) into a hydrophobic groove in Mdm2 (Fig. 5A). Both M2 and Mdm2 showed high similarity to the structure of the parental p53 peptide (from which M2 is derived) bound to Mdm2 (Cα RMSD = 0.43 Å)^19^. A notable difference is a complete one turn extension of the interacting helix in the vGFP-M2 structure comprising the Enhancer residues Val 242, Gln 243, Leu 244 and Val 245 (Fig. 5B). This confirmation is stabilised by the Leu 244 side chain projecting into Mdm2 to more fully occupy its hydrophobic groove.

Comparison with the binary GFP-Enhancer structure^14^ shows highly similar conformations of the GFP and the Enhancer components in vGFP-M2-Mdm2 (Cα RMSD = 0.43 Å). A striking deviation is however seen for residues 1–10 of Enhancer in the two structures (Fig. 5C). In the binary complex, these residues comprise β-strand 1 in the highly conserved β sheet framework observed in variable-domain immunoglobulin folds^30^. In the vGFPE4-M2-Mdm2 complex this region is displaced by up to 30 Å, with the first four amino acids forming part of the extended M2 helix as discussed above. The remaining residues act as a linker connecting to the otherwise structurally conserved Enhancer fold. The integrity of the complex, despite this large translocation attests to the high affinity of the Enhancer for GFP (590 pM)^14^ that is further improved when the two are connected in cis.

### Intracellular targeting using vGFP-scaffolded peptides

**Figure 6:**
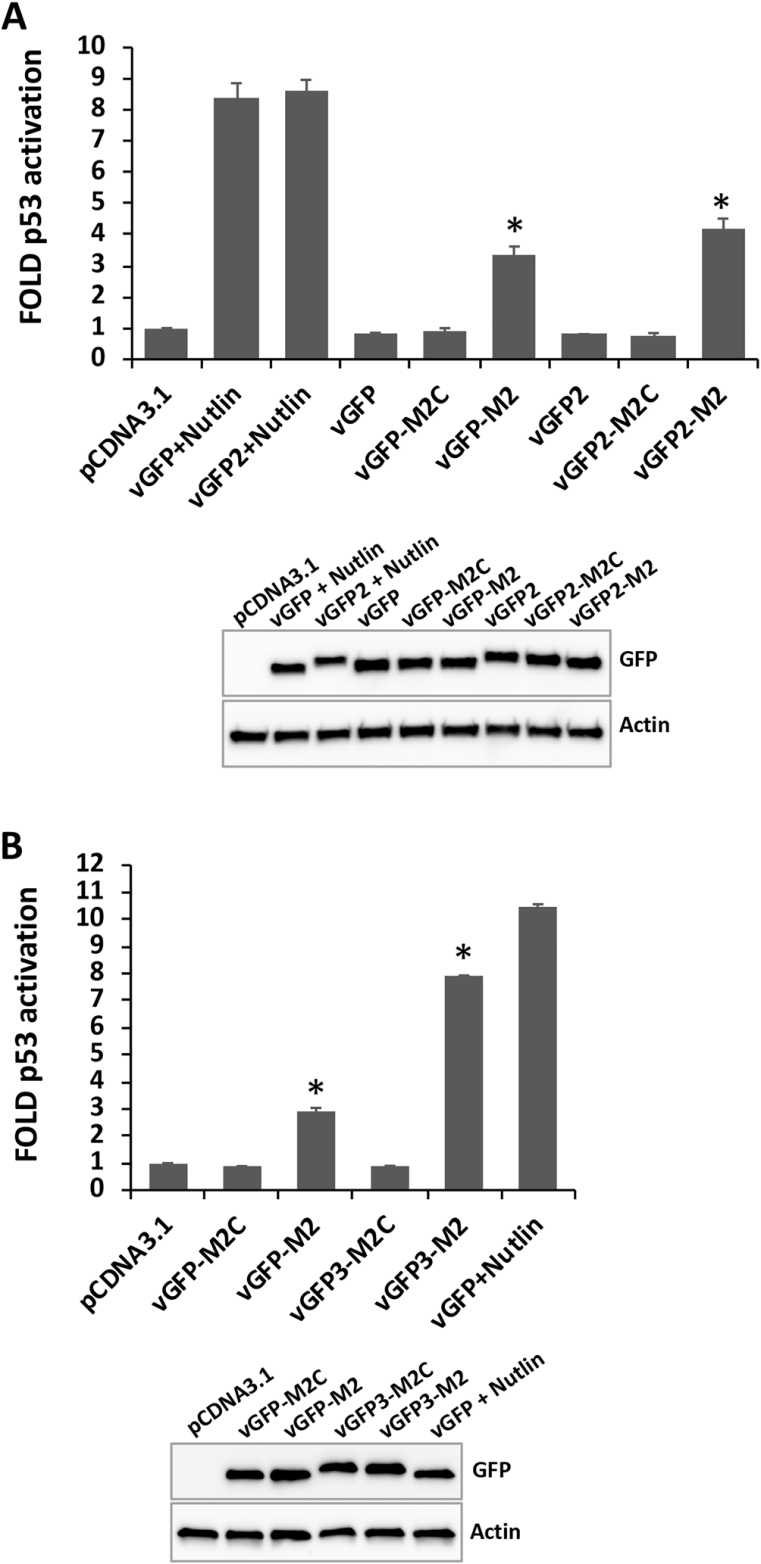
Targeted disruption of the Mdm2-p53 interaction in cells. T22 cells were transfected with indicated vGFP display constructs and p53 reporter gene activity measured as fold over control (pcDNA3.1 vector only). n = 3 ± SD. Small molecule Mdm2 inhibitor Nutlin 3a used as positive control. Respective expression levels of constructs shown in Western blots below. *p < 0.01 compared to vGFP-M2C/vGFP2-M2C/vGFP3-M2C (Student’s t-test).

Next, the intracellular targeting and activity of vGFP-scaffolded peptides was investigated. Inhibition of the p53-Mdm2 protein–protein interaction was measured by transfection of vGFP constructs into T22 cells. This cell line harbours a stably integrated p53-driven β-galactosidase gene that reports on elevated p53 levels arising from Mdm2 inhibition^31,32^. In addition to vGFP-M2 we also tested a construct with an extended linker sequence added C-terminal to the M2 peptide (vGFP2-M2) (Table 1), designed to relieve deformation of the Enhancer domain seen in the Mmd2-bound structure of vGFP-M2 that was designed for biosensing (Fig. 5A). The positive control inhibitor (Nutlin 3a) gave the expected increase of p53 activity in the assay (Fig. 6). Expression of both vGFP-M2 and vGFP2-M2 resulted in clear p53 activation above levels observed for the corresponding vGFP-M2C and vGFP2-M2C controls (Fig. 6A). Activity of vGFP2-M2 was higher than that of vGFP-M2 (41% versus 32% of activity seen for Nutlin 3a positive control). The vGFP-M2-Mdm2 structure indicated that the intramolecularly displaced Enhancer residues 242–245 effectively lengthened the α helical Mdm2-binding interface (Fig. 5B). We therefore made a construct incorporating an additional copy of Enhancer residues 242–246 after the M2 peptide sequence in vGFP2-M2 (vGFP3-M2, Table 1) and measured activity in T22 cells. This iteration notably increased activity over the starting vGFP-M2 construct (75% versus 28% activity seen for Nutlin 3a positive control) (Fig. 6B).

**Figure 7:**
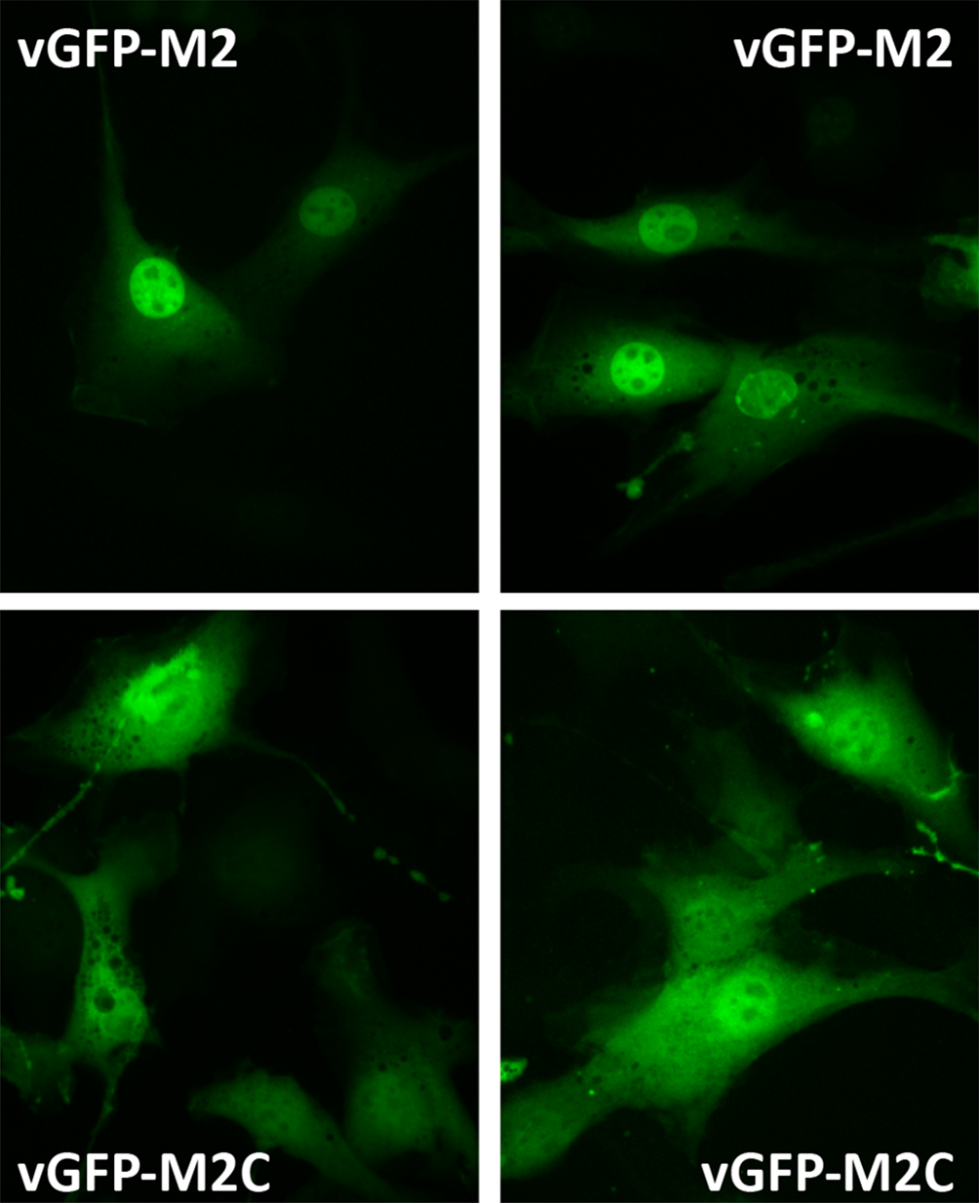
Live cell imaging of T22 cells transfected with plasmids expressing vGFP-M2 (top row) or vGFP-M2C (bottom row). Four z-stacks were taken using a spinning disk confocal fluorescence microscope. Images are projections of sum slices of the z-stacks.

To further confirm cellular target engagement, we imaged live cells transiently expressing vGFP-M2 and vGFP-M2C. vGFP-M2 was mainly present in the nucleus, consistent with the predominantly nuclear location of Mdm2^33^. In contrast, vGFP-M2C localized throughout the cell (Fig. 7, Fig. S4).

**Figure 8:**
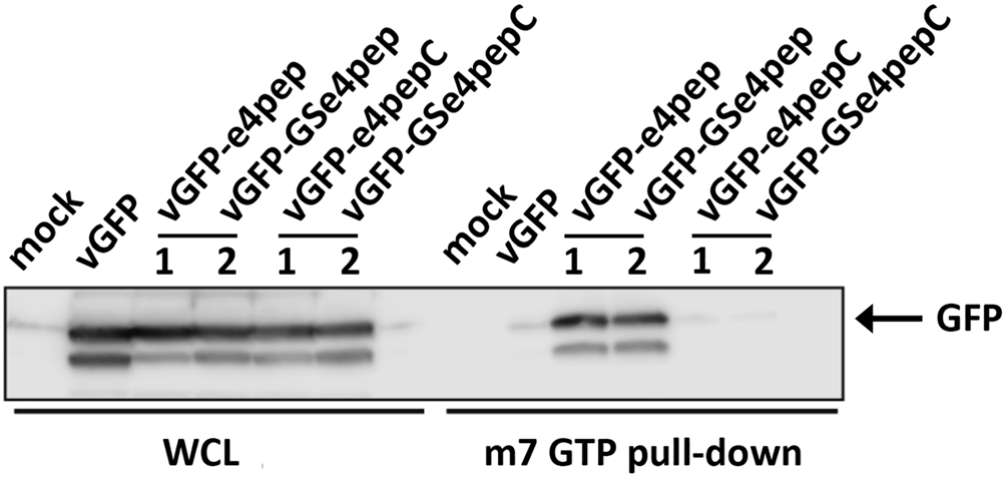
Intracellular targeting of eIF4E by engineered vGFP. HEK293 cells were transfected with indicated constructs and eIF4E complexed proteins isolated by pull-down using m7GTP beads. Immunoblot shows interaction with vGFP-e4pep/vGFP-GSe4pep but not controls vGFP-e4pepC/vGFP-GSe4pepC. *WCL* whole cell lysate.

We further studied vGFP robustness by using it to scaffold a 14 amino acid peptide (e4pep) that binds to the translational initiation factor eIF4E^34^. Targeting constructs (vGFP-e4pep and vGFP-GSe4pep) were transfected into HEK293 cells and assayed for eIF4E engagement by pull-down from lysate using m7GTP beads. vGFP-GSe4pep comprises an additional Gly-Ser motif in the linker region (Table 1). The m7GTP beads specifically bind eIF4E via its cap analog interface, enabling its purification along with any bound proteins. After pull-down, bound vGFP constructs were detected by Western blot using anti-GFP antibody. The results indicate clear binding to eIF4E by the vGFP-e4pep/vGFP-GSe4pep proteins, but not the respective vGFP-e4pepC/vGFP-GSe4pepC controls (Table 1) displaying a non-binding variant of e4pep (Fig. 8).

## Discussion

We have described use of vGFP as a scaffold to present different peptides targeting Mdm2 and eIF4E. Disruption of the p53-Mdm2 protein–protein interaction by vGFP-M2 was observed, leading to increased p53 activity in cells. Structural analysis of vGFP-M2 bound to Mdm2 show the scaffolded M2 peptide adopting the optimal α-helical binding conformation seen in both linear and chemically scaffolded (i.e. stapled) Mdm2 binding peptides^19,35,36^. Furthermore, the structure revealed an extension of the M2 peptide α-helix that interfaces with Mdm2. This was achieved by co-opting the first 4 residues from the N-terminus of the fused Enhancer domain that normally adopt a β-strand conformation. Incorporation of these residues into a vGFP variant designed to reduce deformation upon Mdm2 binding (vGFP3-M2) enhanced cellular activity ~ twofold. It will be interesting to see if this modification enhances Mdm2-targeting stapled peptides being developed for clinical applications ^18,37,38^.

Melt curve analysis of vGFP-M2/M2C highlighted moderate thermostability of the vGFP scaffold. Fluorescence of vGFP-M2C and vGFP-M2 respectively started to diminish at 75 °C and 66 °C, clearly highlighting a destabilizing influence of the hydrophobic ‘FWL’ signature triad present in M2 (Table 1) and high-affinity Mdm-2 binding peptides retaining this motif. The intrinsic fluorescence of vGFP-M2 is also lower than vGFP-M2C (Fig. 1B), further suggesting a destabilizing feature of this peptide. We previously scaffolded the M2 peptide into a bacterial copper oxidase and noted an inhibition of enzyme activity not seen for the control M2C peptide^1^, further highlighting unusual properties of the hydrophobic ‘FWL’ triad. We anticipate vGFP thermostability will prevail upon insertion of other bioactive peptides. When coupled with the relatively high expression yields ($\ge$ 10 mg per litre in *E. coli*) and intrinsic high fluorescence, these features make vGFP a strong candidate to add to the scaffolding tool box.

Both endogenous and engineered variants of GFP have been used as display scaffolds to present peptides in permissible loop regions^39–44^. However, peptide insertion has been reported to destabilise the scaffold, reducing fluorescence and/or causing solubility issues in some cases. The vGFP scaffold potentially mitigates this liability by not directly interfering with the GFP fold and allowing for improved fluorescence/stability due to intramolecular interaction with the Enhancer. However, as shown for the M2 peptide, indirect destabilization can still occur. An interesting possibility would be to increase valency and sample novel display topology space by co-opting a known permissive insertion site in the GFP component of vGFP.

Our efforts at developing a vGFP-based biosensor based on target-dependent intramolecular dissociation of GFP and the Enhancer were met with limited success. The structure of the vGFP-M2-Mdm2 complex revealed the GFP-Enhancer interaction was concomitant with significant disruption of the conformation of β-strand 1 in the Enhancer. Despite introducing four mutations into the Enhancer to reduce affinity for GFP, specific interaction with analyte (Mdm2) could only be discerned in vitro using fluorescence melt curve analysis. Therefore, further rational engineering and/or directed evolution of the GFP-Enhancer binding interface may yield a sensor that works in living cells. Future design iterations could also include use of the companion fluorescence-quenching Minimizer nanobody^14^ to improve signal to noise read-outs.

## Methods

### Constructs

The vGFP cassette from pANT-9L (a kind gift from Dr Swaine Chen) was excised using NdeI and HindIII and ligated into cut pET22b (+) vector. Peptide-displaying vGFP constructs for bacterial expression/purification were subsequently made by inverse-PCR mutagenesis of pET22-vGFP using primers indicated in Supplementary Table S1. vGFP-M2 was made using primer pair 1 and 2. vGFP-M2C was made using primers 3 and 4. vGFP-M2.1 and vGFP-M2.2 were made using primer pairs 5/6 and 5/7 respectively. vGFPE4-M2 with four mutations in the Enhancer domain (S273A, R275A, S299A and F342A) was generated by successive Quickchange mutagenesis (Stratagene) of vGFP-M2 using forward and reverse primer pairs for QC-S273A (primers 8/9), QC-R275A (primers 10/11), QC-S299A (primers 12/13) and QC-F342A (primers 14/15). For the eIF4E interaction assay, vGFP-e4pep and vGFP-GSe4pep were first generated by inverse PCR using primer pairs 16/17 and 16/18 respectively. For mammalian expression of vGFP-e4pep and vGFP-GSe4pep, the constructs were subsequently cloned into EcoRI-XhoI site of pCDNA3.1 vector via infusion PCR (Clontech) using primer pair 19/20 for amplification of inserts and primer pair 21/22 for amplification of pCDNA3.1 vector. The controls vGFP-e4pepC and vGFP-GSe4pepC in pCDNA3.1 vector were then generated via Quikchange using primer-pairs 23/24 and 25/26 respectively. The control encoding vGFP only was cloned into pCDNA3.1 vector via infusion PCR using primer pair 27/28 for amplification of insert and primer pair 29/30 for amplification of pCDNA3.1 vector. For mammalian expression of vGFP-M2C and vGFP-M2, the constructs were cloned into the NheI-EcoRI sites of pCDNA3.1 vector via infusion PCR using primer pair 31/32 for amplification of inserts and primer pair 33/34 for amplification of pCDNA3.1 vector. For vGFP2-M2 and vGFP2-M2C, a (GGGGS)_2_ linker was introduced into the vGFP-M2 or vGFP-M2C vector respectively via inverse PCR using primer pairs 35/36 and 35/37. For vGFP3-M2 and vGFP3-M2C, Enhancer derived residues V242-E246 were added between M2 or M2C peptide and the (G_4_S)_2_ linker in vGFP2-M2/M2C constructs via inverse PCR using primer pairs 38/39 and 38/40 respectively.

### Protein expression and purification

vGFP-M2, vGFP-M2C, vGFP-M2.1, vGFP-M2.2 and vGFPE4-M2 constructs were cloned as fusion proteins with C-terminal 6xHis tags. The constructs were then transformed into *Escherichia coli* BL21(DE3) (Invitrogen) competent cells and grown in LB medium at 37 °C. At OD_600 nm_ of 0.6, the cells were induced at 16 °C overnight with 1 mM IPTG (for vGFP-M2 and vGFP-M2C) or 0.5 mM IPTG (for vGFPE4-M2) before harvesting and lysis by sonication. The cell lysate was clarified and applied to a 5 mL HisTrap column (GE Healthcare) pre-equilibrated in binding buffer (50 mM Tris–HCl pH 8, 500 mM NaCl, 20 mM imidazole, 1 mM DTT), washed and eluted off the column using a linear gradient with elution buffer (50 mM Tris–HCl pH 8.0, 500 mM NaCl, 500 mM imidazole, 1 mM DTT) over 30 column volumes. The eluted fractions containing the protein were then pooled and dialyzed into ion-exchange binding buffer (20 mM Tris pH 8, 1 mM DTT) using a HiPrep 26/10 desalting column. The protein was then loaded onto a 1 mL ion-exchange ResourceQ column (GE Healthcare) pre-equilibrated in ion-exchange binding buffer. The column was washed with binding buffer and bound protein was eluted with a linear gradient in elution buffer (20 mM Tris pH 8, 1 M NaCl, 1 mM DTT) over 60 column volumes. Protein purity was assessed by SDS-PAGE, pooled, buffer exchanged into buffer (50 mM Tris pH 8, 150 mM NaCl, 1 mM DTT) and concentrated using Amicon-Ultra (10 kDa MWCO) concentrator. The purified proteins were then used in the subsequent assays. For vGFP-M2 used for structural studies, vGFP-M2 was further purified by loading onto a Superdex 75 16/60 size exclusion column (GE Healthcare) in gel filtration buffer (50 mM Tris pH 8, 150 mM NaCl, 1 mM DTT). Protein purity was assessed by SDS-PAGE, pooled and concentrated using Amicon-Ultra (10 kDa MWCO) concentrator. vGFP-M2.1 and vGFP-M2.2 were induced at OD_600 nm_ by addition of 1 mM IPTG and expression carried out for 4 h at 37 °C. They were purified using His-GraviTrap columns (GE Healthcare) following manufacturer's protocol. Mdm2 (amino acids 6–125) was cloned as a GST-fusion protein, expressed and purified using affinity chromatography and Resource S cation exchange column as previously described^35^. *G. pallidus* RAPc8 amidase was expressed and purified as previously described^45^.

### Mdm2 (6–125) pull-down assay

The purified vGFP-M2, vGFP-M2C, vGFPE4-M2 proteins (10 μM) were incubated with Mdm2 (6–125) at a molar ratio of 1:9 at 4 °C for 3 h, diluted using 1 × Binding/Wash buffer (1 × TE + 500 mM NaCl). The mixture was then incubated with the Dynabeads His-tag isolation system (Thermo Fisher Scientific) at 4 °C for 30 min. Beads were washed and bound protein was eluted by boiling in SDS buffer and analysed by SDS-PAGE.

### Formation of vGFP-M2 -Mdm2 (6–125) complex for structural studies

Purified vGFP-M2 was incubated with purified Mdm2 (1: 2.5 molar ratio) at 4 °C for 4 h. The protein mixture was then filtered and resolved onto a Superdex 75 16/60 size exclusion column (GE Healthcare) in gel filtration buffer (50 mM Tris pH 8, 150 mM NaCl, 1 mM DTT). Protein fractions were then assessed by SDS-PAGE, pooled and concentrated using Amicon-Ultra (10 kDa MWCO) concentrator (Millipore).

### Crystallization and structure determination

All crystals were grown at 16 °C using the sitting drop vapour diffusion method and clarified by centrifugation before setting up the crystallization trials. MDM2-vGFP-PM2 complex was concentrated to approximately 8.8 mg/mL. Crystals of the complex were grown by mixing the complex with the reservoir solution (25% w/v polyethylene glycol 3500, 100 mM Bis–Tris pH 5.5, 200 mM sodium chloride) in a ratio of 1:1. Single crystals were briefly soaked in mother liquor as cryo-protectant and then flash frozen under liquid nitrogen. Diffraction data were collected up to 3 Å resolution at National Synchrotron Radiation Research Center (NSRRC, Taiwan) at beamline BL13B1 at 1 Å wavelength. Data were indexed, integrated, and scaled with the HKL2000 program package (HKL research). Molecular replacement was performed using 3K1K as a search model for GFP and 4UMN for Mdm2 in PHASER^46^. Data collection and refinement statistics are shown in Supplementary Table S2.

Restrained refinement with TLS was carried out using REFMAC^47^ in the CCP4 suite^48^ and model building was done in COOT^49^. Data collection and refinement statistics are shown in Supplementary Table S2. Structure figures were generated using PyMOL (Delano Scientific LLC). Crystal structure coordinates are deposited in the Protein Data Bank with PDB access code 5WTS.

### Thermal stability assay

vGFP variants (10 µM) were incubated alone or with Mdm2/Amidase (100 µM) and Nutlin compounds (1 mM) at room temperature for 30 min in PBS buffer and placed into 0.2 mL thin-wall PCR tubes. Thermal ramping was carried out on a BioRad CFX96 Real Time System and fluorescence was measured over a temperature range between 35 and 100 °C at 0.5 °C increments.

### P53-reporter gene assay in T22 cells

T22 reporter cells (stably transfected with pRGCd-Fos-lacZ carrying a p53-driven β-galactosidase gene) ^31^ were maintained in Dulbecco’s modified Eagle’s medium (DMEM) with 10% (v/v) fetal calf serum (FCS) and 1% (v/v) penicillin/streptomycin. Transfection was carried out using Lipofectamine 3000 transfection reagent (Thermo Fisher Scientific) according to manufacturer’s instructions. For intracellular targeting activity in T22 reporter cells, luciferase expression plasmid was co-transfected with respective vGFP expression constructs at 1:1 ratio, 1.0 μg total plasmid DNA.

### eIF4E interaction assay in HEK 293 cells

Twenty-four hours prior to transfection, HEK 293 cells (Thermo Fisher Scientific) were seeded at a cell density of 1 × 10^6^ per well of a 6-well plate. Transfections in 6-well plates were performed using Lipofectamine 3000 (Thermo Fisher Scientific) with 0.5 μg of plasmid vectors per well according to the manufacturer’s instructions. After 48 h, cells were lysed and m7GTP pull down were performed as described^50^. Briefly, 300 µg of cell lysates were incubated with 20 μL of m7GTP (Jena Bioscience) agarose beads for 3 h at 4 °C on a rotator. Beads were then washed four times with lysis buffer and vGFP-eIF4E complexes eluted by boiling the beads for 5 min at 95 °C in presence of Laemmli buffer. Samples were resolved by SDS/PAGE and transferred onto PVDF membrane using the Trans-Blot Turbo system (Bio-Rad) according to the manufacturer’s protocol. Blocked membranes were blotted with 3H9 anti GFP antibody (Chromotek) followed by incubaton with the secondary goat anti-rat for 1 h at room temperature. Chemiluminescence was then detected with a Licor system (Licor Biosciences).

### Live cell spinning disk confocal fluorescence microscopy

For live cell imaging, T22 cells grown on 27 mm diameter Nunc glass bottom dish (Thermofisher) were transfected 24 h prior to image acquisition. Images were acquired on Nikon Eclipse Ti inverted microscope, using a 40 × oil immersion objective (NA 1.3). Z-stacks were acquired for all images and sum slices projection was applied to all the stack images using ImageJ Software^51^.

## Supplementary Information

Supplementary Information.

## Data Availability

The datasets generated during and/or analysed during the current study are available from the corresponding author on reasonable request.
