# Supplementary material for: Functional display of bioactive peptides on the vGFP scaffold
Source: Sci Rep. 2021 May 12;11:10127. doi: 10.1038/s41598-021-89421-y (PMC8115314; doi:10.1038/s41598-021-89421-y)
Supplement: Supplementary file 1 — Supplementary Information. [file 41598_2021_89421_MOESM1_ESM.pdf]

Supporting Information for : Functional display of bioactive peptides on the vGFP scaffold.

Sharon Min Qi Chee, Jantana Wongsantichon, Lau Sze Yi, Barindra Sana, Yuri Frosi, Robert C. Robinson and Farid J. Ghadessy*

* E-mail: fghadessy@p53Lab.a-star.edu.sg

p53 Laboratory, A*STAR, 8A Biomedical Grove, Singapore 138648;

Figure S1

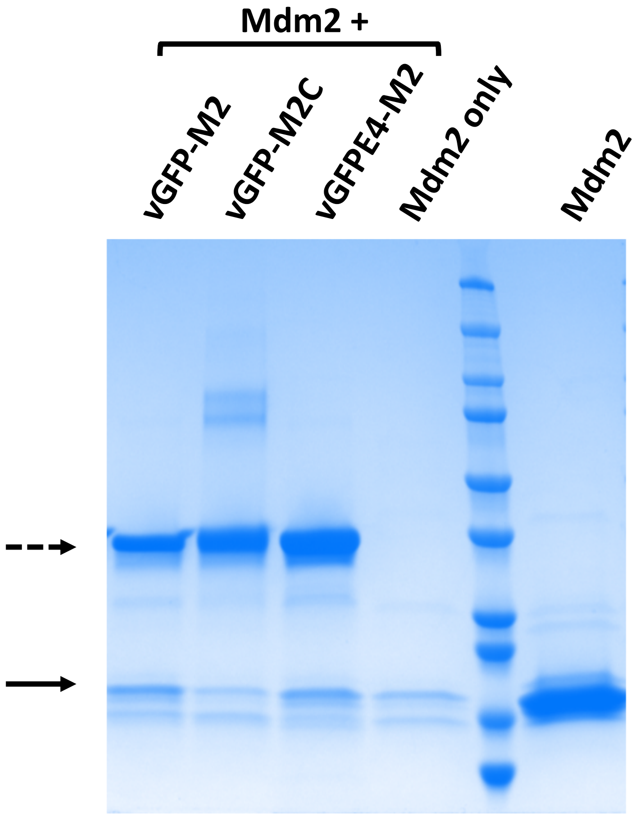

Figure S1. In vitro interaction of vGFP-scaffolded peptides with Mdm2 N-terminal domain (residues 6-125). Indicated purified proteins were (co)-incubated and pulled down complexes analysed by SDS PAGE. Upper and lower arrows respectively correspond to engineered vGFP proteins and interacting Mdm2.

Figure S2

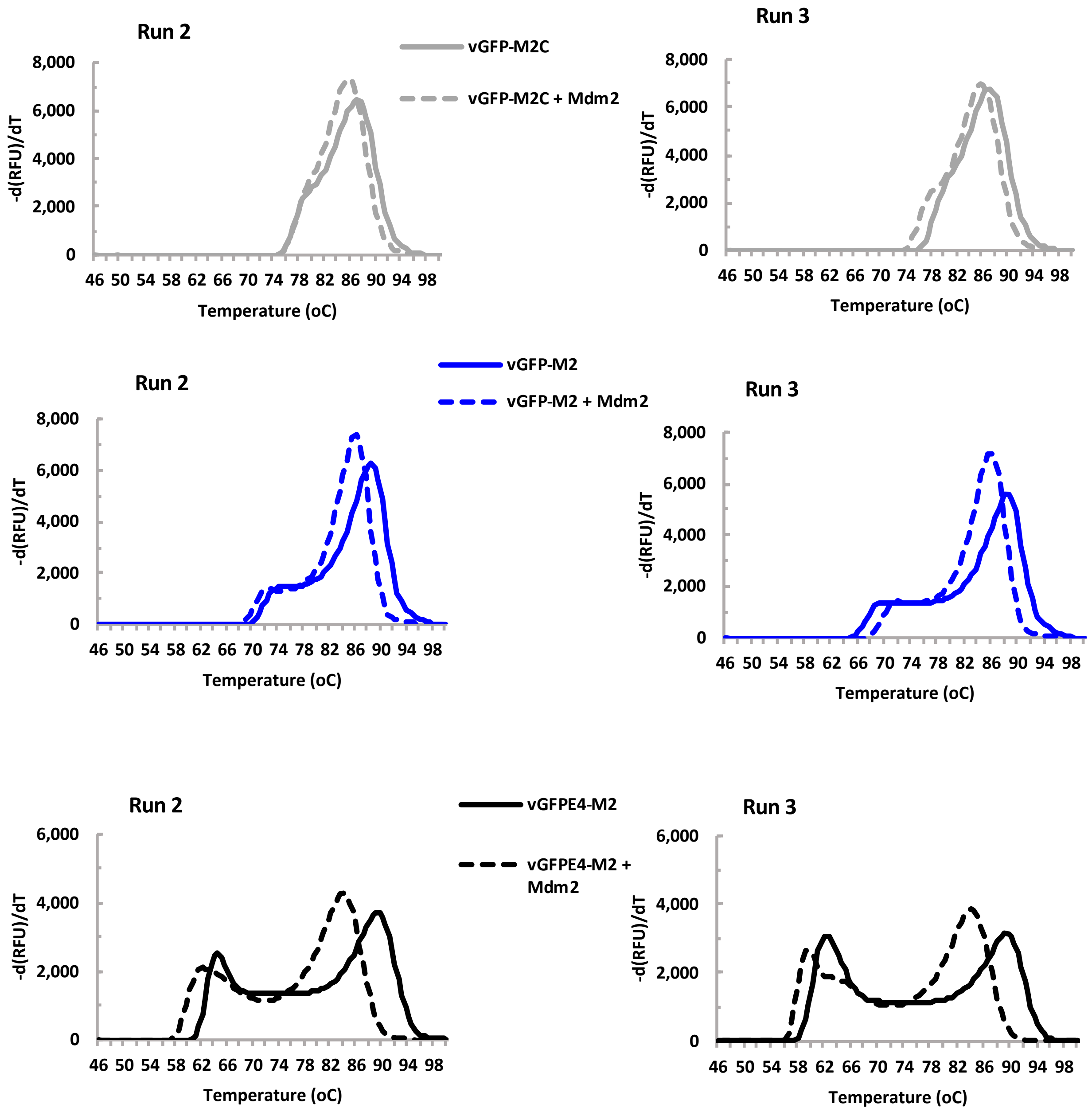

Figure S2. Thermal melt analysis of engineered vGFP proteins. The indicated vGFP variants were incubated alone or with Mdm2 (6-125) and fluorescence measured over shown temperature range.

Figure S3

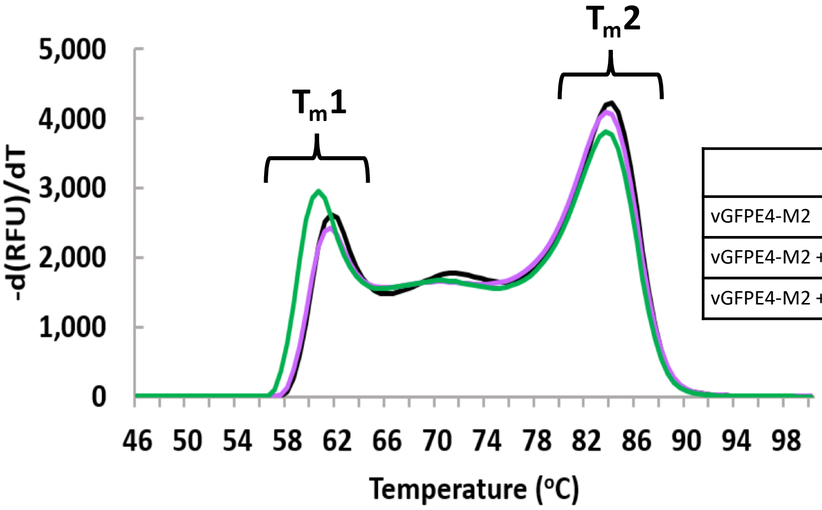

	T_m 1	T_m 2
vGFPE4-M2 (black)	61.33 ± 0.76	84.00 ± 0
vGFPE4-M2 + Nutlin-3a (purple)	61.50 ± 1	83.50 ± 0
vGFPE4-M2 + Nutlin-3b (green)	59.83 ± 0.58	83.50 ± 0

Figure S3. Thermal melt analysis of vGFPE4-M2 alone or in the presence of Nutlin 3a or Nutlin 3b. n=3 ± SD.

Figure S4

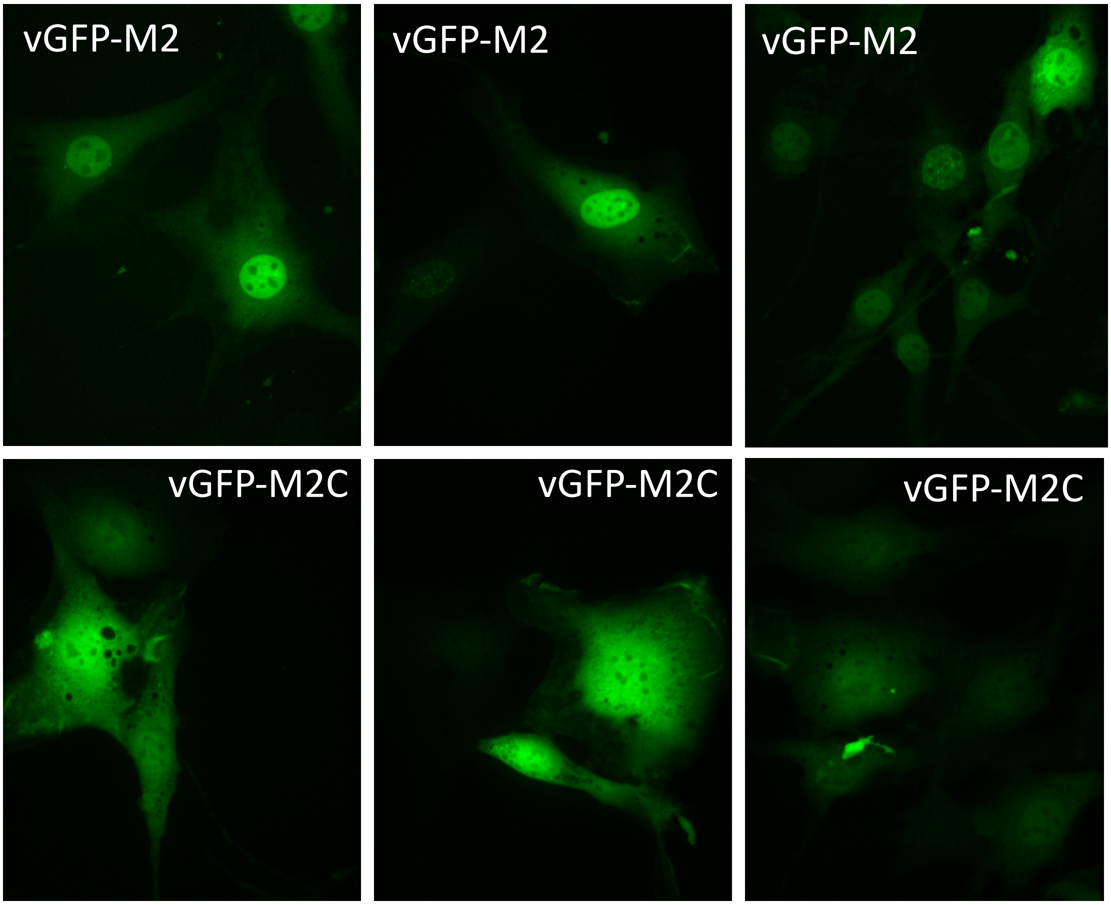

Figure S4. Live cell imaging of T22 cells transfected with plasmids expressing vGFP-M2 (top row) or vGFP-M2C (bottom row). Four z-stacks were taken using a spinning disk confocal fluorescence microscope. Images are projections of sum slices of the z-stacks.

Table S1: Oligonucleotides used in study.

#	Name	Sequence (5' - 3')
1	PMI_F	TTGGGCGCTGCTGAGCGTGCAGTTGGTCGAATCTGGCGGT
2	PMI_R	TATTCCGCAAAGCTGGTTGTAATCCCAGCAGCAGTTACAAACTCAAGAAGGA
3	PMICON_F	TATGCGGCGCTGGCGAGCGTGCAGTTGGTCGAA
4	PMICON_R	TTCCGCCGCGCTGGTTGTAATCCCA
5	PMI-2/3del_F	ACCAGCTTTGCGGAATATTGGGCGCT
6	PMI-2del_R	CCCAGCAGCAGTTACAAACTCAAGAAGGACCA
7	PMI-3del_R	AGCAGCAGTTACAAACTCAAGAAGGACCATGTGGT
8	QC-S273A-F	CTTCCCAGTTAATCGTTATGCGATGCGTTGGTACCGTCAAGCG
9	QC-S273A-R	CGCTTGACGGTACCAACGCATCGCATAACGATTAACTGGGAAG
10	QC-R275A-F	CAGTTAATCGTTATAGCATGGCCTGGTACCGTCAAGCGCCT
11	QC-R275A-R	AGGCGCTTGACGGTACCAGGCCATGCTATAACGATTAACTG
12	QC-S299A-F	TCGGCGGGTGATCGTAGCGCGTACGAAGATTCCGTTAAA
13	QC-S299A-R	TTTAACGGAATCTTCGTACGCGCTACGATCACCCGCCGA
14	QC-vGFP-F342A-F	CAATGTTAATGTGGGTGCGGAATACTGGGGCCAGGG
15	QC-vGFP-F342A-R	CCCTGGCCCCAGTATTCCGCACCCACATTAACATTG
16	InvPCR-EIF-F	TTCCTGATGGAGTGTCGGAACGTGCAGTTGGTCGAATCT
17	InvPCR-EIF-R	TTTCCGGTCATAGATGATCCTTGTAATCCCAGCAGCAGTTACAAACTCAAGAAGGACCAT
18	InvPCR-EIF-GS-R	TTTCCGGTCATAGATGATCCTGCTACCTGTAATCCCAGCAGCAGTTACAAACTCAAGAAGGACCAT
19	INF-vGFP-eIF4E-ins-F	AGTGTGCTGGAATTCATGAGCAAAGGAGAAGAACTTTTCACTGGAGTTG
20	INF-vGFP-eIF4E-ins-R	AGATGCATGCTCGAGTTACTAATGATGATGATGATGATGGGAAACGGTAACTTGCG
21	InvPCR-pCDNA-F	CTCGAGCATGCATCTAGAGGGCCCTATTCTATAG
22	InvPCR-pCDNA-R	GAATTCCAGCACACTGGCGGCCGTTACTAGT
23	QC-eIF4E-CON-F	ATTACAAGGATCATCGCCGACCGGAAATTCGCCGCTGAGTGTCGGAACGTG
24	QC-eIF4E-CON-R	CACGTTCCGACACTCAGCGGCGAATTTCCGGTCGGCGATGATCCTTGTAAT
25	QC-eIF4E-CON-GS-F	AGGTAGCAGGATCATCGCCGACCGGAAATTCGCCGCTGAGTGTCGGAACGTGCAG
26	QC-eIF4E-CON-GS-R	CTGCACGTTCCGACACTCAGCGGCGAATTTCCGGTCGGCGATGATCCTGCTACCT
27	INF-vGFPonly-F	ATGAGCAAAGGAGAAGAACTTTTCACTGGAGTTGTCCCAATTCTTGTTG
28	INF-vGFPonly-R	TGCTCGAGTTACTAATGATGATGATGATGATGGGAAACGGTAACTTGC
29	INF-vGFPonly-pCDNA-F	TAGTAACTCGAGCATGCATCTAGAGGGCCCTA
30	INF-vGFPonly-pCDNA-R	TTCTCCTTTGCTCATGAATTCCAGCACACT
31	I-vGFP-fw	CCCAAGCTGGCTAGCATGAGCAAAGGAGAAGAAC
32	I-vGFP-rev	ATATCTGCAGAATTCTTAATGATGATGATGATGATGGG
33	V-pCDNA-fw	GAATTCTGCAGATATCCAGC
34	V-pCDNA-rev	GCTAGCCAGCTTGGGTCTC
35	vGFP2-GS-fw	GGTGGTGGTGGTTCTGTGCAGTTGGTCGAATCTGGC
36	vGFP2-M2-GS-rev	AGATCCTCCTCCTCCGCTCAGCAGCGCCCAATATTC
37	vGFP2-M2C-GS-rev	AGATCCTCCTCCTCCGCTCGCCAGCGCCGCATATTC
38	VQLVE_GS-fw	GGTAGAGGGAGGAGGAGGATCTGGTGGTG
39	VQLVE_M2-rev	AGTTGAACGCTCAGCAGCGCCCAATATTCC
40	VQLVE_M2C-rev	AGTTGAACGCTCGCCAGCGCCGCATATTCC

Table S2: Data collection and refinement statistics

	vGFP-M2-Mdm2 (6-125)
**Data collection**	
Space group	*I*222
Cell dimensions	
a, *b*, *c* (Å)	51.77, 98.42, 218.99
α, β, γ (°)	90.00, 90.00, 90.00
Resolution (Å)	20.0-3.00 (3.05-3.00)
R_{merge}	0.09 (0.40)
R_{pim}	0.04 (0.19)
$I/\sigma I$	14.2 (2.2)
*CC*1/2	(0.887)
Completeness (%)	97.3 (92.6)
Redundancy	4.7 (4.2)
Unique reflections	11255 (523)
**Refinement**	
Resolution (Å)	20.01-3.00 (3.30-3.00)
No. reflections	10564 (442)
R_{work}	0.204 (0.256)
R_{free}	0.244 (0.285)
Molecules per ASU	2
Residues modeled to each molecule	
A	3-354
B	12-107
No. atoms	3611
Protein	3594
Water	7
B factors	
Protein (chain A/B)	59.1/43.4
Water	26.8
RMSDs	
Bond lengths (Å)	0.002
Bond angles (°)	0.473
**Ramachandran**	
Favored (%)	95.7
Allowed (%)	4.3
Outliers (%)	0

Statistics for the highest resolution shells are shown in parentheses.
$R_{work} = \Sigma_{hkl}||F_{obs}| - k|F_{calc}||/\Sigma_{hkl}|F_{obs}|$.
$R_{free} = \Sigma_{hkl\subset T}||F_{obs}| - k|F_{calc}||/\Sigma_{hkl\subset T}|F_{obs}|$ where *T* represents test set comprising 5% of all reflections excluded during refinement.

Uncropped image files

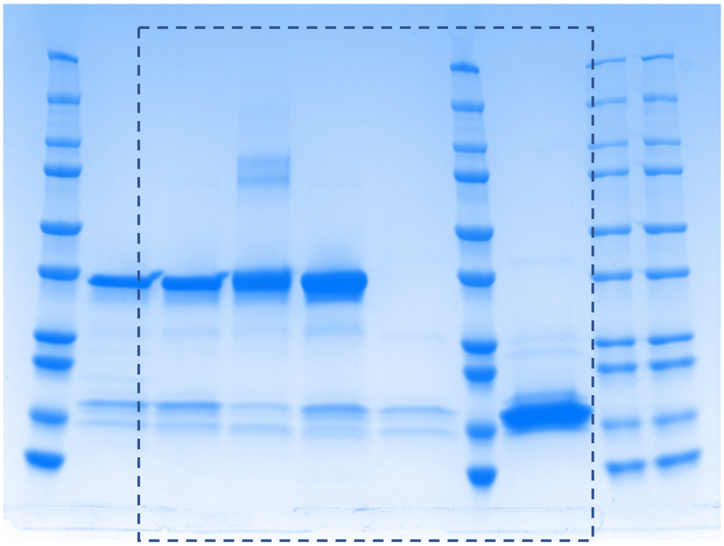

Uncropped image : Fig S1.

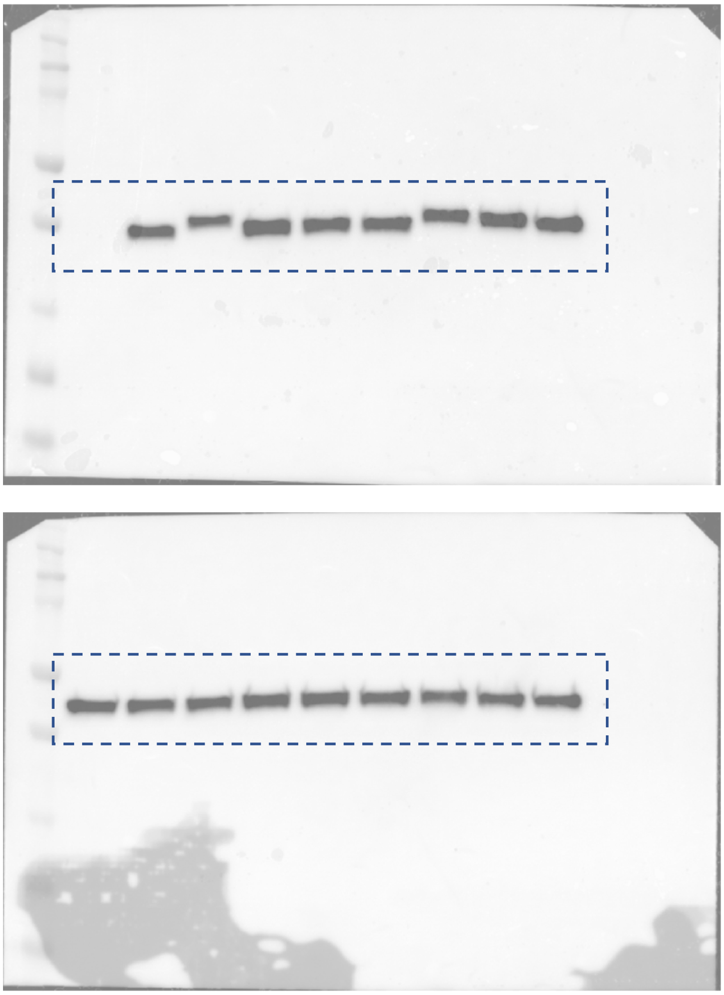

Uncropped images : Fig 6A.

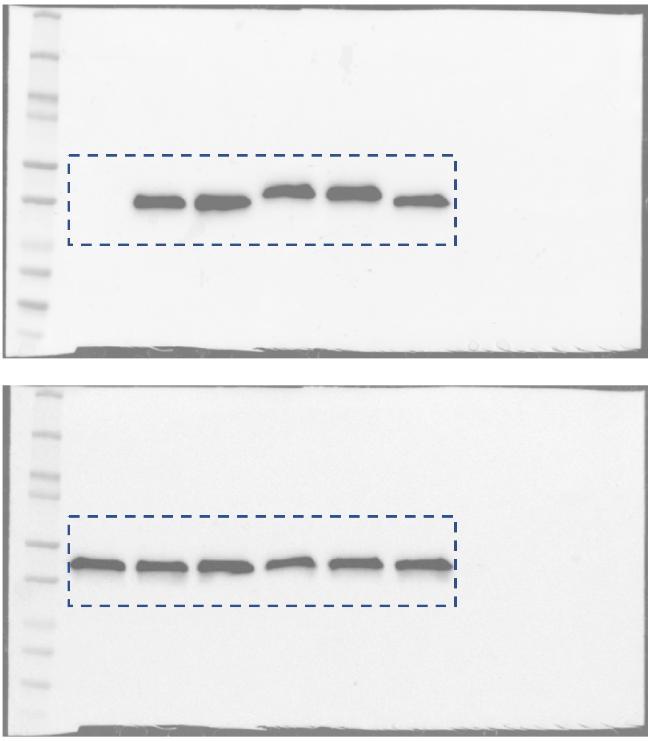

Uncropped images : Fig 6B.

Uncropped image files

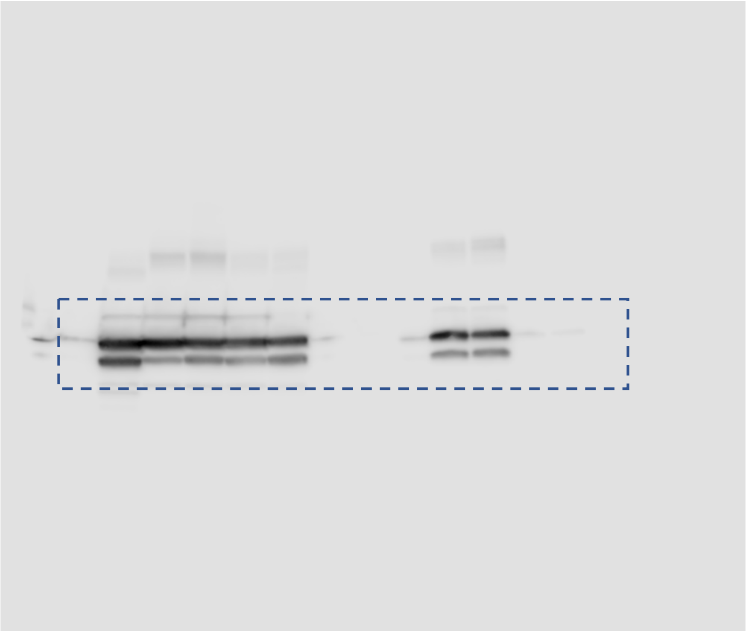

Uncropped image : Fig 8.
